# The usefulness of isometric protocol for foot flexors and extensors in assessing the effects of 16-week rehabilitation regiment in poststroke patients

**DOI:** 10.1186/s12938-019-0678-6

**Published:** 2019-05-14

**Authors:** Ewa Chlebuś, Agnieszka Wareńczak, Margaret Miedzyblocki, Przemysław Lisiński

**Affiliations:** Department of Rehabilitation and Physiotherapy Rehabilitation, University of Medical Sciences, 28 Czerwca 1956 Str., No 135/147, 60-545 Poznan, Poland

**Keywords:** Stroke, Paretic foot, Isometric protocol, Rehabilitation

## Abstract

**Background:**

Ankle joint function in a paretic limb has a fundamental impact on mobility. Return of joint function is a measure of early poststroke physical rehabilitation. This study aims to assess the suitability of using the isometric protocol for objective evaluation of flexor and extensor muscle strength in the paretic limb of poststroke patients.

**Methods:**

34 patients (F: 9, M: 25) aged 51–79 years with hemiparesis following an acute ischemic stroke and 34 healthy controls were examined using the isometric protocol measured on the Biodex System^®^. The following parameters were analyzed: peak torque [PT], average torque [AVGT], average torque/body weight [AVGT/BW] for flexors and extensors, and AVGT flexor/AVGT extensor [agonist/antagonist ratio] of the paretic foot, the nonparetic foot and foot of healthy controls using three foot–shank positions (15°, 0°, and − 15°) prior to rehabilitation commencement and at its completion 16 weeks later.

**Results:**

Prior to rehabilitation commencement, nonparetic foot differed significantly (*p* < 0.05) from healthy foot controls in all parameters and all positions for flexors and in all positions for foot–shank positions of 0° and − 15° for extensors. At rehabilitation program completion the following parameters increased significantly for the paretic foot: PT, AVGT, and AVGT/BW for foot extensors in all tested positions, and PT for foot flexors in foot–shank position of − 15°. The nonparetic foot however, showed no significant difference following rehabilitation regardless parameter or foot position tested for flexors and extensors alike. Prior to rehabilitation agonist/antagonist ratio in the paretic foot differed significantly from corresponding parameter in the control group for the foot–shank positions of 15° and 0°, whereas at rehabilitation completion, the two groups showed significant difference only in foot–shank position of 0°.

**Conclusions:**

In the early period following stroke, there is a significant strengthening of the paretic limb, but no improvement in the strength of nonparetic limb.

## Background

According to literature, ankle joint function in a paretic limb has a fundamental impact on mobility and is vital to improving patients’ quality of life following stroke [1, 2]. Therefore this study will focus on evaluating ankle joint efficiency as a major factor determining gait efficiency. The return of joint function is a measure of early poststroke rehabilitation effectiveness.

Ankle joint efficiency of a healthy limb is determined by several factors including range of motion, dependent primarily on tendo-muscular unit elasticity [3], the intactness of the proprioceptive pathway, responsible for "deep sensation” within the ankle joint [4] the strength of individual muscles as well as relationships between forces generated by antagonistic muscle groups [5, 6]. To date, the relationship between these determinants (of ankle joint efficiency) has not been clearly defined [7, 8]. The method for measuring strength becomes an issue when one takes into account the strength of the muscles controlling the movement of the ankle [9].

Subject literature reveals that the isokinetic protocol is generally used to assess the strength of flexor and extensor muscle groups [10, 11]. Strength assessment using this protocol depends largely on preserved range of motion within the ankle joint. However, many patients in the early poststroke period are unable to generate adequate force to produce ankle motion, due to progressing spasticity of the tendo-muscular units [12]. This reduces the effectiveness of the isokinetic protocol in assessing the muscle strength of foot flexors and extensors in poststroke patients. Therefore, it seems that only the isometric protocol allows for an objective assessment of paretic muscle strengths, regardless of the degree of joint flexibility or the speed of joint movement [13, 14]. Moreover, studies which used the isokinetic protocol focused only on the paretic foot. There are no studies which compare the muscle strength of the paretic as well as the nonparetic foot to that of healthy controls.

### Objectives

To assess the suitability of using the isometric protocol for an objective evaluation of foot flexor and extensor muscle strength in the early poststroke period.

To compare the flexor and extensor muscle strength of the paretic foot to that of the nonparetic foot and to the foot of healthy controls in three test positions.

To determine whether the implementation of lower limb rehabilitation following stroke improves the muscular strength of both the paretic foot and the nonparetic foot in hemiparetic patients.

## Materials and methods

This was an observational study with a control group. The study was carried out between January 2015 and December 2017 at the Neurological Rehabilitation Department of Wiktor Dega Orthopedic-Rehabilitation Clinical Hospital, Poznań University of Medical Sciences.

The study group consisted of 34 patients with post ischemic stroke hemiparesis. There were nine women and 25 men examined, with an average age of 65.1 (range 51–79, SD: 8.59). Nineteen patients had a right-sided hemiparesis and remaining 15 patients—left-sided hemiparesis. The control group consisted of 34 healthy volunteers with no prior history of trauma or neurological disease affecting the structure and function of the ankle joint. This group included 13 women and 21 men with an average age of 61.6 years (range 53–75, SD: 5.59). Table 1 shows patient characteristics for both the study and the control group.

**Table 1:** Comparison of the general characteristics patients before and after rehabilitation

Characteristics	Ischemic stroke group	Control group	*p*
	*n* = 34	*n* = 34	
Gender (male/female)	25/9	21/13	
Age (years)	64.9 ± 9.2	61.6 ± 5.6	> 0.05
Height (cm)	171.3 ± 9.6	171.6 ± 9.7	> 0.05
Weight (kg)	81.5 ± 13.9	80.2 ± 15.8	> 0.05
Body mass index (kg/m^2^)	27.9 ± 4.9	27.2 ± 4.6	> 0.05
Risk factors			
Smoke	12 (35.29%)	10 (29.41%)	
Diabetes	10 (29.41%)	8 (23.52%)	
Arterial hypertension	30 (88.23%)	25 (73.52%)	
Atrial fibrillation	15 (44.11%)	6 (17.64%)	
Time since stroke (days)	15 ± 2		
Time of rehabilitation treatment (days)	125 ± 11		
Affected side			
Left	15 (44.2%)		
Right	19 (55.8%)		
Localization (Bamford classification)			
TACI	4 (11.76%)		
PACI	16 (47.05%)		
LACI	12 (35.29%)		
POCI	2 (5.8%)		
AMTS	9.67 ± 0.47		
NIHSS before rehabilitation	12 ± 4		
NIHSS after rehabilitation	9 ± 4		
Barthel Index before rehabilitation	41.05 ± 19.2		
Barthel Index after rehabilitation	73.60 ± 19.8		
Modified Rankin Scale (*n*): I/II/III before rehabilitation	15/15/4		
Modified Rankin Scale (*n*): I/II/III after rehabilitation	2014-12-08		
Modified Ashworth Scale (*n*): grade 1/1+ before rehabilitation	18/16		
Modified Ashworth Scale (*n*): grade 1/1+ after rehabilitation	24-paź		
Brumstrom stages of lower limb (*n*): IV/V/VI before rehabilitation	8/22/4		
Brumstrom stages of lower limb (*n*): IV/V/VI after rehabilitation	4/18/12		

Values are presented as mean ± SD or number (*n*) of the patients (%)

TACI, total anterior circulation syndrome; PACI, partial airculation circulation syndrome; LACI, lacunar airculation circulation syndrome; POCI, posterior airculation circulation syndrome; AMT, abbreviated mental test score; NIHSS, National Institutes of Health Stroke Scale

The inclusion criteria were as follows:

1. Lower limb paresis, with unilateral hemiparesis, resulting from the first episode of ischemic stroke
2. Low spasticity in the examined foot (scores of 1 or 1+ in Modified Ashworth Scale—MAS)
3. Selective motor impairment in the ankle joint of the paretic foot (stage IV and higher in Brumstrom Stages of Lower Limb)
4. Ability to generate muscle strength in foot–shanks positions during the biomechanical examination on the Biodex System^®^
5. Patients in logical verbal contact, able to follow commands (AMTS > 9).

Patients with following conditions were excluded from the study:

1. Tetraparesis
2. Multiple stroke episodes
3. Lower limb paresis resulting from etiology other than stroke
4. Cognitive impairment and aphasia (AMTS < 9)
5. Prior injury of the tested ankle joint
6. Foot clonus.

A written consent was obtained from each patient participating in the study prior to study commencement and following a detailed description of the study and assessment techniques. The approval for the use of the Biodex device in the study was obtained from the Bioethical Commission of the Medical University of Poznan (Approval No. 539/18).

### Biomechanical examination

The examination of patients with the Biodex System Pro 4, Biodex Medical Systems, INC. was conducted prior to rehabilitation commencement, on average 5 weeks (35.32 ± 2.88 days) poststroke, and again after 16 weeks of rehabilitation on the day of hospital discharge. Control group was examined once. The test involved performing a maximal contraction of foot flexors followed by a maximal contraction of foot extensors, without any actual limb movement. Each participant (from study and control group) completed the task three times, alternately contracting the flexors and extensors of the tested foot by applying pressure on the dynamometer’s footplate Each muscle contraction was carried out for a duration of 5 s, with a 5 s break between each new contraction to allow for full muscle relaxation.

### Study protocol

During the examination, the patient was seated in an armchair which was connected to the electronic dynamometer. Patient’s torso was stabilized with two belts running across each other and over the patient’s chest, whereas the upper limbs were placed on arm supports allowing muscles to loosen. The examined lower limb was positioned on an attachment which offloaded the hip and knee joint simultaneously maintaining both joints in 70° of flexion. The foot was propped up with an attachment allowing maximum relaxation of extensor and flexor muscles of the examined foot.

The procedure first focused on the nonparetic limb. Prior to each examination, the researcher established the range of motion in the examined ankle joint. Examination commenced with the dynamometer locking the foot in 15° of flexion. In this position, the patient maximally contracted foot flexors for 5 s. This contraction was followed by a 5 s break, and then a 5 s maximal contraction of foot extensors. Each test was carried out in a series of three alternating flexions and extensions. After completing the series there was a 15 s break to allow for relaxation of the examined muscles. The above protocol was then carried out for the foot locked into a neutral position (0°) and, following completion, for the foot locked into 15° of extension. Finally, a report was printed out presenting individual results for the three foot–shank positions. In order to avoid interpretative mistakes, we adopted the following reference terms for the test positions: 15° **→ **flexion, 0° **→ **neutral position, − 15° **→ **extension.

### Outcome measures

The isometric protocol was used in obtaining the following parameters: peak torque [PT]—the single highest value of the moment of force recorded, average torque [AVGT]—average value of the moment of force calculated over the course of entire test and AVGT/BW—average value of the moment of force in relation to the subject’s body weight, for both extensors and flexors of the paretic foot, nonparetic foot and foot of healthy volunteers from the control group. The tests were carried out for three foot–shank positions: 15° in flexion, 0° in neutral position, 15° in extension, prior to rehabilitation commencement and again at its conclusion 16 weeks later. These foot–shank positions were determined in accordance with the test protocol specified by the manufacturer of the Biodex device (http://www.biodex.com). In addition, the relationship between the AVGT values for flexors and extensors for all examined feet, in three tested positions, and both before and after rehabilitation were examined.

### Protocol for poststroke rehabilitation of the foot

All patients underwent the standard comprehensive neurological rehabilitation program commonly used in foot rehabilitation for patients in the early poststroke period [15].

Individual, minor differences in the rehabilitation program pertained to the duration of individual therapeutic sessions and the intensity of the exercises, which resulted from differences in the patients’ mobility. The rehabilitation treatment was based on Proprioceptive Neuromuscular Facilitation (PNF) using the standard for the foot: flexion, adduction, external rotation based on the contract–relax technique. The rehabilitation program included, in each case, 15 min-long exercises stretching flexor muscles of the paretic foot and 20-min-long isotonic exercises for strengthening of both lower limbs. With the increasing patient’s mobility, additional 25-min-long balance and coordination exercises, including gait re-education were introduced. Electrostimulation of foot extensor muscles was the most commonly employed method from the available physical therapy methods. The total duration of rehabilitation was 16 weeks, 2 h a day, 6 days a week (Monday to Saturday).

### Statistical analysis

Data were analyzed with the Statistica version 13.1. Demographic data and clinical characteristics are presented as means, standard deviations (SD) and median. The Shapiro–Wilk test was used to assess the normality of the distributions in the test score. A paired *t* test or Wilcoxon’s signed ranks test were used to analyze the rehabilitation effects within the stroke group (pre- and postintervention). The one-way ANOVA or nonparametric Kruskal–Wallis test were used to analyze differences between the paretic, nonparetic and the healthy foot of control group volunteers. Post hoc comparisons were performed to find at which parameters and at which positions differences between groups were significant. *p* values less than 0.05 were considered statistically significant.

## Results

### Foot extensor torque

Analyzing the values of the parameters describing the strength of the paretic foot extensors (PT, AVGT, AVGT/BW), we noticed the significant increase of their values after rehabilitation, in all the tested positions (*p* < 0.05). However, the values of all parameters for the paretic limb were significantly different in all positions compared to the same values for healthy feet of controlled group, both before and after rehabilitation. The results of the nonparetic limb compared to the control group healthy limbs differed significantly after the therapy for positions 0° and − 15° for PT and AVGT/BW parameters (Table 2).

**Table 2:** Comparison of parameters foot extensor torque between the groups

Isometric parameter	Peak torque (NM)			Avg peak TQ (NM)			Avg PT/BW (%)		
Position	(+)15°	0°	(−)15°	(+)15°	0°	(−)15°	(+)15°	0°	(−)15°
Paretic side									
Before									
Mean ± SD	14.6 ± 9.6	10.4 ± 6.8	4.9 ± 2.9	12.8 ± 9.0	9.3 ± 6.3	4.6 ± 2.7	16.2 ± 11.4	11.8 ± 8.0	5.8 ± 3.2
Median	13.2	9.2	5.3	11.1	8.6	4.9	14.6	10.8	6.1
After									
Mean ± SD	19.6 ± 10.7	14.9 ± 8.1	8.0 ± 5.5	17.3 ± 9.7	13.6 ± 7.7	7.5 ± 5.1	21.8 ± 12.3	16.7 ± 9.8	9.3 ± 6.4
Median	20.4ª	15.7ª	6.6ª	18.7ª	12.5ª	6.4ª	22.2ª	16.1ª	7.7ª
Non paretic side									
Before									
Mean ± SD	21.6 ± 11.7	17.4 ± 10.4	8.2 ± 5.5	18. ± 11.1	15.7 ± 10.0	7.3 ± 5.0	23.8 ± 15.1	20.1 ± 13.6	9.5 ± 7.0
Median	21.0	15.1	6.5	17.2	12.8	5.3	19.0	13.9	6.9
After									
Mean ± SD	23.3 ± 11.9	18.6 ± 9.4	10.7 ± 7.1	20.2 ± 10.9	17.0 ± 8.9	11.4 ± 10.9	25.3 ± 14.0	21.5 ± 12.0	12.7 ± 9.6
Median	23.7	18.2	8.8	18.8	16.7	8.6	24.2	20.4	9.9
Control group									
Mean ± SD	28.9 ± 14.5	24.1 ± 9.2	14.8 ± 7.1	26.2 ± 14.1	21.4 ± 9.2	13.7 ± 6.9	33.3 ± 16.9	27.8 ± 10.9	17.6 ± 8.2
Median	30.0	22.2	14.4	26.5	18.1	12.5	33.3	25.5	16.4
Before rehabilitation *p* value									
Paretic vs non paretic									
*p*	NS	*0.012*	NS	NS	*0.011*	NS	NS	*0.027*	NS
Non paretic vs control									
*p*	NS	*0.013*	*<* *0.001*	NS	*0.026*	*<* *0.001*	NS	*0.007*	*<* *0.001*
Paretic vs control									
*p*	*<* *0.001*	*<* *0.001*	*<* *0.001*	*<* *0.001*	*<* *0.001*	*<* *0.001*	*<* *0.001*	*<* *0.001*	*<* *0.001*
After rehabilitation *p* value									
Paretic vs non paretic									
*p*	NS	NS	NS	NS	NS	NS	NS	NS	NS
Non paretic vs control									
*p*	NS	*0.035*	*0.046*	NS	NS	NS	NS	*0.047*	*0.014*
Paretic vs control									
*p*	*0.008*	*<* *0.001*	*<* *0.001*	*0.02*	*0.002*	*<* *0.001*	*0.005*	*<* *0.001*	*<* *0.001*

*NS* no significant difference, *p* > 0.05

ªSignificant difference between pre- and post intervention outcomes

### Foot flexor torque

While comparing the values of parameters measuring the strength of flexors of the paretic foot (PT, AVGT, and AVGT/BW) before and after the therapy, a significant difference was observed only for the PT parameter at position − 15°. Significant differences in PT, AVGT, AVGT/BW parameters were also noted, both for paretic and nonparetic feet compared to the values of the same parameters for healthy subjects in all three examined positions, both before and after rehabilitation. On the other hand, there were no significant differences between the results of the paretic and nonparetic limbs for both series of measurements before and after rehabilitation (Table 3).

**Table 3:** Comparison of parameters foot flexor torque between the groups

Isometric parameter	Peak torque (NM)			Avg peak TQ (NM)			Avg PT/BW (%)		
Position	(+)15°	0°	(−)15°	(+)15°	0°	(−)15°	(+)15°	0°	(−)15°
Paretic side									
Before									
Mean ± SD	18.3 ± 14.9	27.3 ± 24.5	33.6 ± 32.2	15.4 ± 14.4	23.8 ± 22.0	31.1 ± 30.5	19.2 ± 17.5	29.7 ± 26.5	38.5 ± 36.9
Median	14.2	17.4	22.4	10.1	15.2	19	12.8	18.6	22.8
After									
Mean ± SD	20.5 ± 17.2	32.4 ± 26.8	42.0 ± 33.0	17.6 ± 16.5	28.7 ± 25.5	36.7 ± 30.6	22.0 ± 19.4	36.0 ± 30.3	45.9 ± 37.0
Median	14.5	26.6	33.6ª	11.7	22.8	29	14.7	27.1	33.8
Non paretic side									
Before									
Mean ± SD	26.4 ± 18.7	38.8 ± 28.6	47.6 ± 38.7	21.7 ± 15.8	33.7 ± 25.0	42.0 ± 35.8	27.2 ± 19.4	42.2 ± 31.6	51.7 ± 44.7
Median	22.7	36.5	34.9	20.1	31.6	27.8	24.7	36.2	31.5
After									
Mean ± SD	28.9 ± 23.2	42.5 ± 35.3	51.1 ± 37.3	23.9 ± 21.7	39.0 ± 34.3	48.7 ± 39.6	30.5 ± 24.9	48.3 ± 40.3	59.5 ± 45.8
Median	22.9	34.9	38.7	17.1	31.6	34.4	21.8	35.1	43
Control group									
Mean ± SD	47.0 ± 20.9	78.2 ± 33.2	107.3 ± 44.7	42.5 ± 19.8	72.9 ± 32.4	99.9 ± 43.7	52.8 ± 20.8	90.2 ± 32.5	121.3 ± 41.9
Median	42.5	70.8	104	38.7	66	99.9	52.9	92.8	128.2
Before rehabilitation *p* value									
Paretic vs non paretic									
*p*	NS	NS	NS	NS	NS	NS	NS	NS	NS
Non paretic vs control									
*p*	*<* *0.001*	*<* *0.001*	*<* *0.001*	*<* *0.001*	*<* *0.001*	*<* *0.001*	*<* *0.001*	*<* *0.001*	*<* *0.001*
Paretic vs control									
*p*	*<* *0.001*	*<* *0.001*	*<* *0.001*	*<* *0.001*	*<* *0.001*	*<* *0.001*	*<* *0.001*	*<* *0.001*	*<* *0.001*
After rehabilitation *p* value									
Paretic vs non paretic									
*p*	NS	NS	NS	NS	NS	NS	NS	NS	NS
Non paretic vs control									
*p*	*0.001*	*<* *0.001*	*<* *0.001*	*<* *0.001*	*<* *0.001*	*<* *0.001*	*<* *0.001*	*<* *0.001*	*<* *0.001*
Paretic vs control									
*p*	*<* *0.001*	*<* *0.001*	*<* *0.001*	*<* *0.001*	*<* *0.001*	*<* *0.001*	*<* *0.001*	*<* *0.001*	*<* *0.001*

*NS* no significant difference, *p* > 0.05

ªSignificant difference between pre- and post intervention outcomes

### Agonist/antagonist ratio

The assessment of the AVGT flexor/AVGT extensor ratio showed a significant difference between the paretic foot before rehabilitation and the foot of healthy group in positions 15° and 0°. After the rehabilitation, significant differences were observed between the paretic and the healthy foot for position 0° only and between the nonparetic and the healthy foot in positions 15° and 0° (Table 4).

**Table 4:** Comparison of parameters agonist/antagonist ratio between the groups

Isometric parameter	Agon vs antagon (%)		
Position	(+)15°	0°	(−)15°
Paretic side			
Before			
Mean ± SD	101.8 ± 55.2	58.4 ± 32.3	25.8 ± 23.0
Median	87.2	55.2	20.8
After			
Mean ± SD	101.5 ± 62.7	59.5 ± 39.3	33.7 ± 49.4
Median	98.4	55.7	22.6
Non paretic side			
Before			
Mean ± SD	92.0 ± 49.0	55.5 ± 51.3	26.6 ± 27.7
Median	92.3	39.9	19.9
After			
Mean ± SD	123.3 ± 83.8	62.0 ± 41.6	33.2 ± 45.8
Median	95.7	51.8	18.4
Control group			
Mean ± SD	69.3 ± 25.3	36.2 ± 15.1	16.0 ± 7.1
Median	63.9	33.2	14.9
Before rehabilitation *p* value			
Paretic vs non paretic			
*p*	NS	NS	NS
Non paretic vs control			
*p*	NS	NS	NS
Paretic vs control			
*p*	*0.027*	*0.003*	NS
After rehabilitation *p* value			
Paretic vs non paretic			
*p*	NS	NS	NS
Non paretic vs control			
*p*	*0.004*	*0.011*	NS
Paretic vs control			
*p*	NS	*0.014*	NS

## Discussion

Limb muscle weakness contralateral to the damaged brain hemisphere is one of the most common symptoms of stroke [16]. In this study, we demonstrated that with smaller "initial" muscle stretch at initiation of contraction (extensors in the position of − 15° and flexors in the position of 15°), these muscles generated lower force expressed by PT, AVGPT, and AVGPT/BW (Tables 2, 3). Ada et al. [17] showed that the muscle strength deficit in stroke patients is in inverse proportion the length of the muscle at the moment of initiation of contraction (shorter length was related to greater muscle insufficiency). Whereas Maynard et al. [18] proved that the reduction in extensor strength impacts mainly gait speed, while inefficient flexors are responsible for poor spatial coordination.

Novak and Brouwer [10] found out that the poststroke patients had lower value for the muscle strength of the paretic lower limb compared to the nonparetic limb and limb of healthy people at similar age. This observation is consistent with the results of our research, in which we also showed significant differences in the values of the analyzed parameters for extensors and flexors between the paretic foot, nonparetic foot and healthy foot (Tables 2, 3). This proves that contrary to the general believe muscle weakness in patients with diagnosed poststroke hemiparesis, affects not only a paretic side but also the other half of the body theoretically considered as asymptomatic or fully functional. Therefore, the assessment of muscle strength of both lower limbs may be a useful indicator of gait function recovery after stroke.

Most often the isokinetic protocol [19] and very rarely isometric protocol [13] are used in order to assess the strength of the paretic muscles. In the course of their research, while attempting to determine the normative values of the maximum force tested with the use of Biodex System 3 PRO, Harbo et al. [11] came to the conclusion that the values obtained in tests depend on the type of protocol used (isokinetic or isometric) and vary in reference to age, height, and weight of a patient performing the test. Certain aspects of our study regarding the assessment of AVGPT/BW value before and after rehabilitation did not show the dependence of AVGPT values on the subjects' body weight (Tables 2, 3).

A majority of the published research regarding poststroke mobility deficiency in hemiplegics contains results of strength measurements of paretic foot extensors and flexors in the late period after stroke with the use of isokinetic protocol. The examples of such procedure are studies conducted by Hsu et al. [20]. Conversely, in our work, we evaluated extensor and flexor tensions of the paretic foot in patients in the early period after stroke using an isometric protocol expressed by PT, AVGPT, and AVGPT/BW. This can be considered a new approach as we did not find examples of similar studies in the available literature. The choice of the isometric protocol was dictated by the early poststroke period, in which patients usually cannot generate enough force that would translate into movement. In addition, in isokinetic protocol, the test is carried out using predetermined axial rotation speed, and, as we determined in the pilot studies, patients are unable to complete such tests because rotations are too fast for them. What's more, attempts to adjust to the set speed often caused pain which prevented further processing. This methodological limitation of the isokinetic protocol was also highlighted in studies by Gray et al. [21].

Most studies in the available literature concentrated on the effects of exercises strengthening only paretic foot extensors in the late after-stroke period, with reference to the extensors of healthy persons [22–25]. In all the studies mentioned, the authors determined that the strength of the paretic foot extensors is significantly lower than the strength of the extensors of healthy foot, which negatively affects the patient's mobility. In our work, we also demonstrated that before and after the rehabilitation (conducted in the same way for all patients), PT, AVGPT, and AVGPT/BW values for paretic foot significantly differed from the values for healthy feet, which could seemingly prove the lack of poststroke rehabilitation of the foot.

However, on comparing the values of these parameters measured for the paretic and nonparetic foot extensors, we found that after rehabilitation they did not differ significantly, if only for the neutral (0°) position of the foot in relation to the shin. In our opinion, this is a good prognosis which encourages future modifications to the rehabilitation treatment which we studied because it indicates the achievement, to some extent, of symmetry in tension of foot extensors in both limbs of the hemiplegic. Hence, there is need to develop more effective methods to strengthen these muscles in the early poststroke period, which will be the subject of our future research.

We believe that an isolated assessment of the paretic foot extensors does not provide a full view of the degree of the patient’s limitations in terms of the ability to control movement in the ankle and possible gait impairment. Hence, in our study, we proposed joint assessment of extensors and flexors of the foot and both lower limbs of hemiplegics. The similar approach has been presented by Chisholm et al. [26] and Kitatani et al. [27] who also emphasized the importance of incorporating methods of comprehensive improvement of antagonistic muscles into the rehabilitation program and stressed/pointed out on the need to conduct further research in this direction. The rationale for the comprehensive treatment, according to these researchers, is the sequential activation of antagonistic muscles in gait (more effective propulsion and more effective foot extension in the transfer phase).

According to Jiang et al. [28], patients after stroke are characterized by reduced dynamics of coactivation of paretic foot flexors and extensors, especially during the extension which leads to balance disturbances during the support phase of gait. In the literature, we found only a few studies assessing the decrease in strength of the paretic foot flexor and its effects on the patient’s mobility [1, 5, 29]. The results of our research (Table 3) indicate that in patients in the early poststroke period occur not only significant weakening of the paretic foot flexor but also of the nonparetic foot, compared to healthy feet of controlled group; the phenomenon which up to this point has not been reported in the publications. In contrast to the results achieved for foot extensors (Table 2), PT, AVGPT, and AVGPT/BW values for the paretic foot did not significantly differ from the ones for nonparetic foot, both in pre- and postrehabilitation tests. However, similar to the case of extensors, the values of these parameters were significantly different for both paretic foot and nonparetic foot in comparison/compared with feet of healthy people, in tests before as well as after rehabilitation (Table 3). This calls into question, even more than in the case of extensors, the effectiveness of rehabilitation procedures used to strengthen flexor muscles of the feet. We plan to conduct future studies, in which we will test several other types of exercises which are used for strengthening of foot flexors [30–35], in order to determine the most effective in poststroke rehabilitation.

Interesting conclusions, in our opinion, result from the comparison of AVGT flexor/AVGT extensor ratio (Table 4); the analysis of which has not been so far presented in the literature.

The values obtained in our study show that after the completion of rehabilitation, the aforementioned relation tested on the paretic foot in the − 15° position did not differ from the results of tests performed on healthy person's foot, which may indicate that the evaluateds in our work of functionality improvement treatment were successful, at least for this position.

## Conclusion

In the early period following stroke, there is not only a significant weakening of the paretic limb, but also that of the nonparetic limb. Accurate and applicable in the early poststroke period, the isometric protocol is a useful tool for verifying the effects of rehabilitation, by measuring changes in PT, AVGT, and AVGT values for flexor to extensor muscles of the foot.

## Data Availability

Not applicable.

## Abbreviations

PT: peak torque
AVGT: average torque
AVGT/BW: average torque/body weight
TACI: total anterior circulation syndrome
PACI: partial airculation circulation syndrome
LACI: lacunar airculation circulation syndrome
POCI: posterior airculation circulation syndrome
AMT: abbreviated mental test score
NIHSS: National Institutes of Health Stroke Scale
NS: no significant
